# Evaluation of serum TWEAK levels and treatment response in psoriasis and psoriatic arthritis: a prospective comparative case–control study of adalimumab and methotrexate

**DOI:** 10.1007/s10067-026-07981-8

**Published:** 2026-03-18

**Authors:** Eisa M. Hegazy, Eslam Abdelfattah Sadek, Shimaa Saber Ahmed

**Affiliations:** 1 Department of Dermatology, Venereology, and Andrology, Faculty of Medicine, Qena University, Qena, Egypt; 2 Department of Public Health and Community Medicine, Faculty of Medicine, Qena University, Qena, Egypt; 3 Rheumatology and Rehabilitation Department, Faculty of Medicine, Luxor University, Luxor, Egypt

**Keywords:** Adalimumab, DAPSA, Methotrexate, PASI, Psoriatic arthritis, TWEAK

## Abstract

**Introduction:**

Psoriasis vulgaris and psoriatic arthritis (PsA) are chronic immune-mediated diseases with systemic impact. Tumor necrosis factor (TNF)–related weak inducer of apoptosis (TWEAK) is a pro-inflammatory cytokine implicated in immune regulation and tissue remodeling, but its role in psoriatic disease remains incompletely understood.

**Methods:**

This prospective case–control study included 100 subjects: 30 psoriasis patients receiving adalimumab, 30 PsA patients receiving methotrexate, and 40 healthy controls. Serum TWEAK was measured by ELISA at baseline and after 24 weeks. PASI and DAPSA scores were evaluated at both visits.

**Results:**

Baseline TWEAK was higher in psoriasis (3.85 ± 0.62 ng/mL) and PsA patients (4.12 ± 0.71 ng/mL) than in controls (1.95 ± 0.54 ng/mL, *p* < 0.001). After 24 weeks, TWEAK decreased in both the adalimumab (2.21 ± 0.49 ng/mL) and methotrexate groups (2.67 ± 0.53 ng/mL, *p* < 0.001). Clinical improvement assessed by PASI and DAPSA paralleled this biochemical reduction, suggesting that TWEAK may serve as a biomarker of treatment response. TWEAK showed a stronger correlation with PASI after 24 weeks of treatment than with DAPSA.

**Conclusion:**

Elevated TWEAK reflects psoriasis and PsA activity. Adalimumab and methotrexate reduce TWEAK alongside clinical improvement, supporting its potential as a biomarker for treatment monitoring.

**Table:** 

**Key Points** *• Serum TWEAK levels were substantially elevated in psoriasis and psoriatic arthritis patients in comparison to controls.* *• TWEAK levels decreased following treatment with methotrexate and adalimumab.* *• Clinical improvement and disease activity scores were positively correlated with a decrease in TWEAK levels.* *• TWEAK may be a valuable biomarker for the monitoring of treatment response in psoriasis.*

## Introduction

Psoriasis is a chronic, immune-mediated skin condition with a global prevalence of 2–3% [1]. Psoriatic arthritis (PsA), an inflammatory arthropathy involving joints, entheses, and the axial skeleton, develops in approximately one-third of psoriasis patients and significantly impairs quality of life [2].

It is characterized by keratinocyte hyperproliferation, abnormal immune activation, and systemic inflammation. Psoriatic arthritis (PsA) develops in up to 40% of patients, a heterogeneous inflammatory arthropathy affecting peripheral joints, entheses, and the axial skeleton [2]. Both conditions have a significant impact on quality of life and are correlated with an excess risk of cardiovascular, metabolic, and mental comorbidities.

Recent advances have highlighted the role of cytokine networks in psoriatic pathogenesis. The TNF-related weak inducer of apoptosis (TWEAK) has been implicated in inflammatory diseases [3–5, 10]. TWEAK is a member of the TNF superfamily that acts through the fibroblast growth factor–inducible 14 (Fn14) receptor to promote inflammation, angiogenesis, and tissue remodeling [3, 4]. TWEAK has been demonstrated to play a role in autoimmune disease; however, limited data exist regarding its dual role in cutaneous and joint manifestations of psoriatic disease [5, 10, 12, 15, 17].

Biologic agents like adalimumab, a monoclonal antibody against TNF-α, and conventional disease-modifying antirheumatic drugs (DMARDs) like methotrexate are standard therapies for moderate-to-severe psoriatic disease [6, 7]. Their effects on TWEAK modulation, however, have not been well studied.

This study aims to evaluate serum TWEAK levels before and after therapy with adalimumab or methotrexate and correlate them with both PASI (for skin) and DAPSA (for joint) indices.

## Patients and methods

### Study participants and design

This prospective comparative case–control research was conducted at a tertiary referral hospital between June 2024 and June 2025. Ethical approval has been obtained (IRB No. DVA021/4/204/4/844). Written informed consent was obtained from all participants prior to enrollment. The psoriasis group received adalimumab, and the PsA group received methotrexate, reflecting the real-world therapeutic standards.

#### Sample size calculation

Prior to data collection, an a priori sample size calculation was performed based on the primary outcome of interest (TWEAK levels). For comparison of means across three groups (controls, psoriasis, and psoriatic arthritis), the sample size per group was calculated using the formula for one-way ANOVA:

$$n\:=\:((k-1)\ast(Z\_alpha/2\:+\:Z\_beta{)^\wedge{2}}\ast sigma^\wedge{2})\;/\;(k\ast Delta^\wedge{2})$$

where

*n = number of participants per group, * *k* = number of groups (3), * sigma^2 = expected variance of TWEAK levels, * Delta = expected difference between group means. *Z_alpha/2 = critical value for significance level alpha = 0.05 (two-sided), * Z_beta = critical value for power = 0.80 (Z_beta ~ 0.84).

Based on prior literature reporting significant differences in TWEAK between diseased and control subjects [5], a large effect size was assumed (Cohen’s *f* = 0.40). Accordingly, a minimum of 20 participants per group was required. Our planned recruitment included 40 controls, 30 psoriasis patients, and 30 PsA patients, ensuring sufficient statistical power (> 95%) to detect significant differences in TWEAK levels between groups.

The psoriasis group received adalimumab, and the PsA group fulfilling CASPAR criteria received methotrexate, reflecting the real-world therapeutic standards. Adalimumab is recommended for moderate-to-severe psoriasis unresponsive to topical or conventional systemic agents, whereas methotrexate remains the first-line DMARD for PsA in Egypt due to cost and accessibility. The inclusion of two therapeutic arms was intended to reflect real-world treatment standards.

**Fig. 1:**
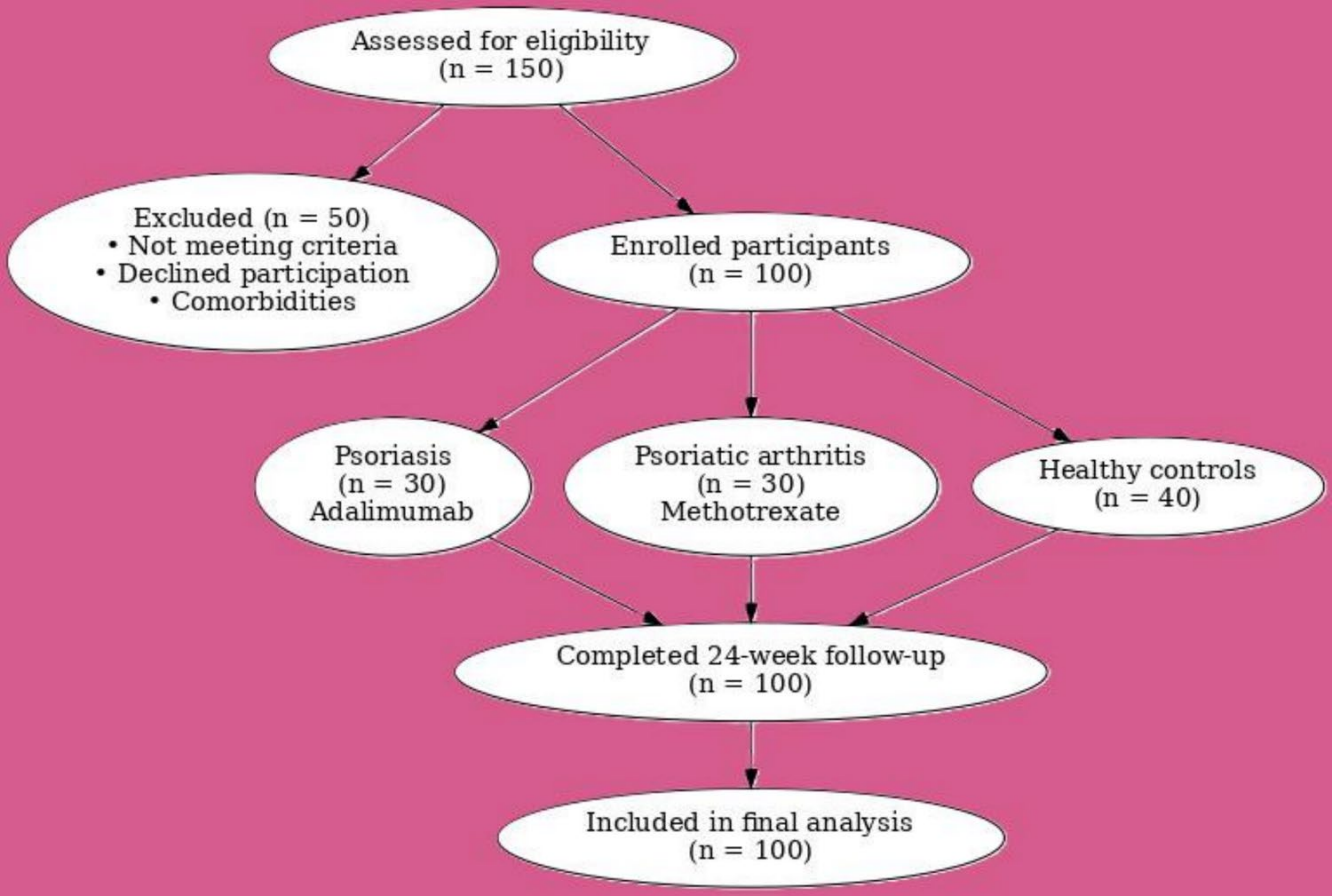
CONSORT-style flow diagram illustrating patient enrollment, allocation, follow-up, and final analysis

A systematic patient flow diagram was developed to depict patient screening, eligibility, allocation, follow-up, and analysis. Despite the study’s lack of randomization, a CONSORT-style flow diagram was employed to improve transparency and clarity in the reporting of participant progression through the study phases (Fig. 1).

- Inclusion criteria: Adults 18–65 years old with chronic plaque psoriasis or PsA, naïve to systemic therapy for ≥ 12 weeks.
- Exclusion criteria: Pregnancy, systemic infections, hepatic or renal insufficiency, hepatitis B/C infection, tuberculosis.

#### Clinical assessment

- PASI: evaluated in the psoriasis group at baseline and 24 weeks

Psoriasis severity was measured using the Psoriasis Area and Severity Index (PASI). PASI calculation consists of two major steps: calculating the body surface area (BSA) covered with lesions and the assessment of the lesions’ severity. The affected area and lesion characteristics generate a score from 0 to 72. The amount of disease (BSA covered with lesions) is estimated by determining what percentage of the skin on a person’s body is affected, with the size of the palm of the hand equal to about 1% of the skin. The body of the patient is divided into four sections, each scored by itself: the head and neck correspond to 10%, the upper extremities 20%, the trunk 30%, and the lower extremities 40% of the total body surface. Within each area, the lesion’s severity is estimated by erythema (redness), induration (thickness), and desquamation (scaling); the severity of each parameter is noted on a scale from 0 (none) to 4 (maximum). The sum of all three severity parameters is then calculated for each section of skin, multiplied by the area score for that area, and then multiplied by the weight of the respective Sect. (0.1 for head, 0.2 for arms, 0.3 for body, and 0.4 for legs) [20].

- DAPSA: evaluated in the PsA group at baseline and 24 weeks

The Disease Activity Index for Psoriatic Arthritis (DAPSA) was used to systematically measure the activity of psoriatic arthritis. DAPSA is a validated composite outcome measure that combines objective and patient-reported criteria. The 68 tender joint count, the 66 swollen joint count, the serum C-reactive protein concentration (mg/dL), the patient’s global assessment of disease activity, and the patient’s estimate of pain severity were all used to figure out DAPSA. The last two were reported on a 0–10 visual analogue scale. This multidimensional index offers a dependable measurement of inflammatory joint burden and overall disease activity in psoriatic arthritis, and it has been extensively utilized in both clinical trials and standard treatment [21].

### Laboratory tests

- Serum TWEAK measured by ELISA at baseline and week 24

TWEAK is a soluble cytokine belonging to the TNF superfamily and can be easily measured in serum using standardized ELISA-based assays [5]. Although TWEAK has a relatively short biological half-life, its serum levels reflect continuous immune activation, supporting its use as a marker for monitoring disease activity and treatment response over longitudinal follow-up periods [5].

- Routine investigations (CBC, liver/renal function, lipid profile, hepatitis, and TB screening)

### Treatment regimens

- Adalimumab: 80 mL subcutaneously at week 0, 40 mL at week 1, then 40 mL every 2 weeks.
- Methotrexate: 15–25 mg/week subcutaneously or orally with folic acid supplementation.

The present study intentionally excluded combination therapy with methotrexate and adalimumab. Patients received treatment in accordance with real-world clinical practices in our environment, where biologic monotherapy or traditional DMARD monotherapy is generally favored due to financial limitations and safety concerns. This method facilitated a more lucid understanding of the distinct impacts of each therapy modality on serum TWEAK levels.

### Statistical analysis

Analyses performed using SPSS v26. Descriptive statistics have been done for quantitative information as mean ± SD (standard deviation) for normally distributed quantitative data, while it has been done for qualitative information as number and percentage. Inferential analyses were done for quantitative parameters utilizing the Shapiro–Wilk test for normality testing. The chi-square test was used for comparison between two pieces of qualitative information. One-way ANOVA [analysis of variance] has been utilized to compare the means of more than 2 groups with a parametric distribution after performing a test of normality. The paired samples *t*-test has been utilized to determine the significant variance among the means of two related groups. Correlation analyses were performed using Pearson’s correlation coefficient.

A *p*-value < 0.05 was considered statistically significant.

## Results

There were no statistically significant differences between the control, psoriasis, and PsA groups in terms of age, gender distribution, or BMI (*p* < 0.05). Serum TWEAK levels were significantly higher in both psoriasis and PsA patients compared to controls (4.01 ± 0.61 ng/mL and 4.10 ± 0.68 ng/mL vs 1.98 ± 0.50 ng/mL, *p* < 0.001) (Table 1).

**Table 1:** Baseline characteristics of study participants

Variable		Controls (*n* = 40)	Psoriasis (*n* = 30)	PsA (*n* = 30)	*p*-value
Age (years, mean ± SD)		41.2 ± 7.8	42.5 ± 8.1	43.1 ± 6.9	0.62
Gender *n* (%)	Male	24 (60%)	19 (63%)	20 (67%)	0.74
	Female	16 (40.0%)	11 (36.7%)	10 (33.3%)	
BMI (kg/m^2^, mean ± SD)		27.1 ± 3.5	27.5 ± 3.6	27.8 ± 3.2	0.81
TWEAK levels (baseline)		1.977 ± 0.50	4.01 ± 0.61	4.10 ± 0.68	< 0.001^**^
Tender joint count		–	–	24.30 ± 8.27	–
Swollen joint count		–	–	8.00 ± 5.11	–
PASI score (baseline)		–	18.6 ± 5.1	–	–
DAPSA score (baseline)		–	–	18.73 ± 5.06	–

^*^Chi-square test was used to compare proportions. **ANOVA test was used to compare quantitative data; *statistically significant *p* value < 0.05

At baseline, the mean PASI score in psoriasis patients was 18.6 ± 5.1, while the mean DAPSA score in PsA patients was 18.73 ± 5.06. After 24 weeks of treatment, serum TWEAK levels significantly decreased in both treatment groups. In psoriasis patients treated with adalimumab, TWEAK levels dropped from 3.85 ± 0.62 ng/mL to 2.21 ± 0.49 ng/mL (*p* < 0.001). Similarly, in PsA patients treated with methotrexate, levels declined from 4.12 ± 0.71 ng/mL to 2.67 ± 0.53 ng/mL (*p* < 0.001) (Table 2).

**Table 2:** Comparison of TWEAK levels at baseline and after 24 weeks of treatment between studied groups

Group	TWEAK levels		*p*-value
	Baseline (ng/mL)	Week 24 (ng/mL)	
Controls (*n* = 40)	1.95 ± 0.54	–	–
Psoriasis + adalimumab (*n* = 30)	3.85 ± 0.62	2.21 ± 0.49	< 0.001^*^
PsA + methotrexate (*n* = 30)	4.12 ± 0.71	2.67 ± 0.53	< 0.001^*^

Paired samples *t*-test, *statistically significant *p* value < 0.05

**Fig. 2:**
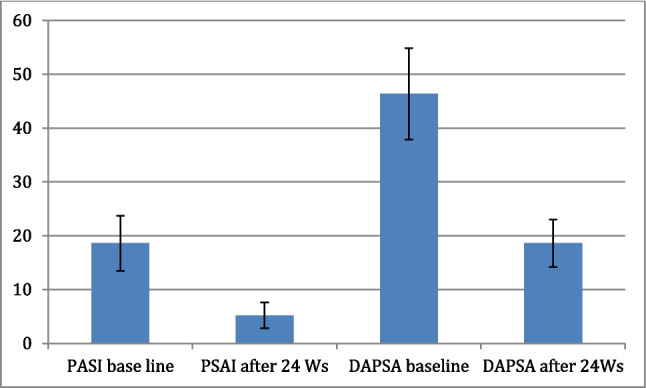
Mean ± SD of PASI (psoriasis cohort) and DAPSA (PsA cohort) before and after 24 weeks of treatment

Clinical disease activity scores also showed significant improvement following treatment (Fig. 2). The mean PASI score decreased from 18.6 ± 5.1 to 5.2 ± 2.4 in psoriasis patients (*p* < 0.001), and the DAPSA score dropped from 46.37 ± 8.49 to 18.59 ± 4.45 in PsA patients (*p* < 0.001) (Table 3).

**Table 3:** Changes in PASI and DAPSA scores before and after treatment between psoriasis and PsA pts

Group	Baseline	After 24 week of ttt	*p*-value
(PASI score) in psoriasis pts (*n* = 30)	18.6 ± 5.1	5.2 ± 2.4	< 0.001^*^
(DAPSA score) in PsA pts (*n* = 30)	46.37 ± 8.49	18.59 ± 4.45	< 0.001^*^

Paired samples *t*-test, *statistically significant *p* value < 0.05

No significant gender-related differences were observed in treatment response.

Both male and female patients demonstrated comparable reductions in serum TWEAK levels, PASI, and DAPSA scores (*p* > 0.05) (Table 4).

**Table 4:** Effect of gender on treatment response in terms of TWEAK, PASI, and DAPSA scores in psoriasis and PsA pts

Variables		Gender		PV within gender	(Improvement over time × gender interaction
		Male	Female		
TWEAK *n* = 60		4.052 ± 0.68	4.06 ± 0.60	0.936	*F* = 0.190, pV= 0.664
		2.46 ± 0.91	2.4 ± 0.75		
PASI score) in psoriasis pts (*n* = 30)	Baseline	17.46 ± 6.14	4.76 ± 2.39	0.169	*F* = 1.20, pV = 0.282
	After 24 Ws of ttt	20.45 ± 5.31	5.96 ± 2.45		
(DAPSA score) in PsA pts (*n* = 30)	Baseline	46.32 ± 7.45	46.47 ± 10.72	0.941	*F* = 0.017, pV = 0.896
	After 24 Ws of ttt	18.73 ± 5.06	18.33 ± 3.12		

Repeated measures ANOVA; *F*, statistical *F*; *n*, number; *Ws*, weeks; *ttt*, treatment

**Fig. 3:**
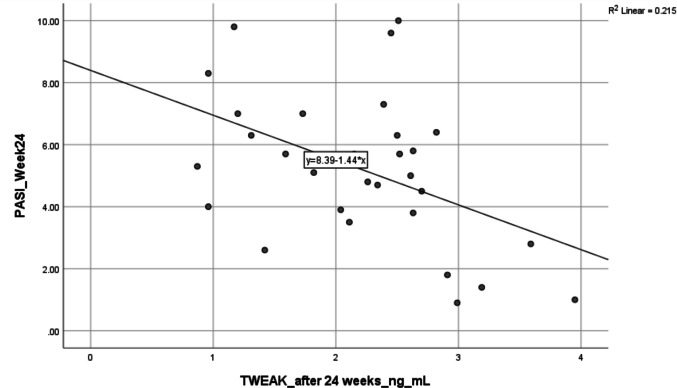
Correlations between TWEAK levels at baseline after week 24 and PASI scores after week 24 in PsA patients

**Fig. 4:**
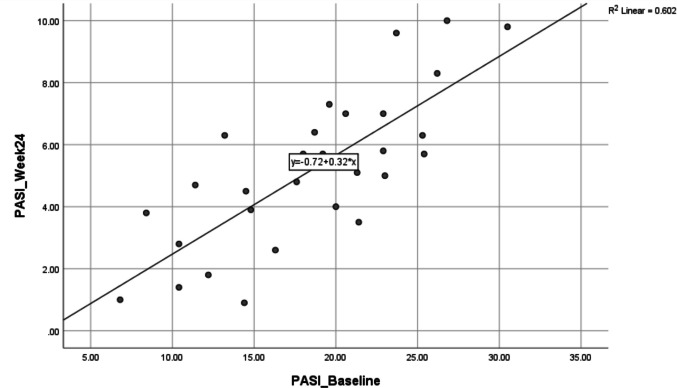
Correlations between PASI scores at baseline and week 24 in psoriasis patients

**Fig. 5:**
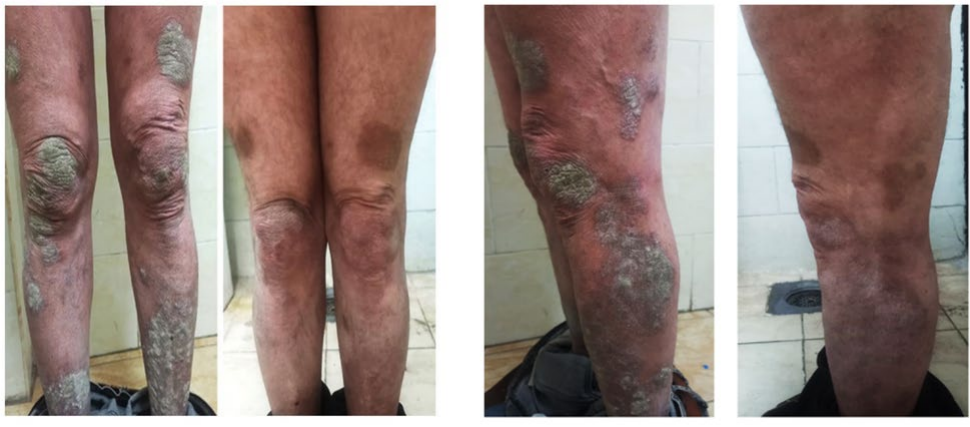
Representative case on adalimumab before and after therapy

**Fig. 6:**
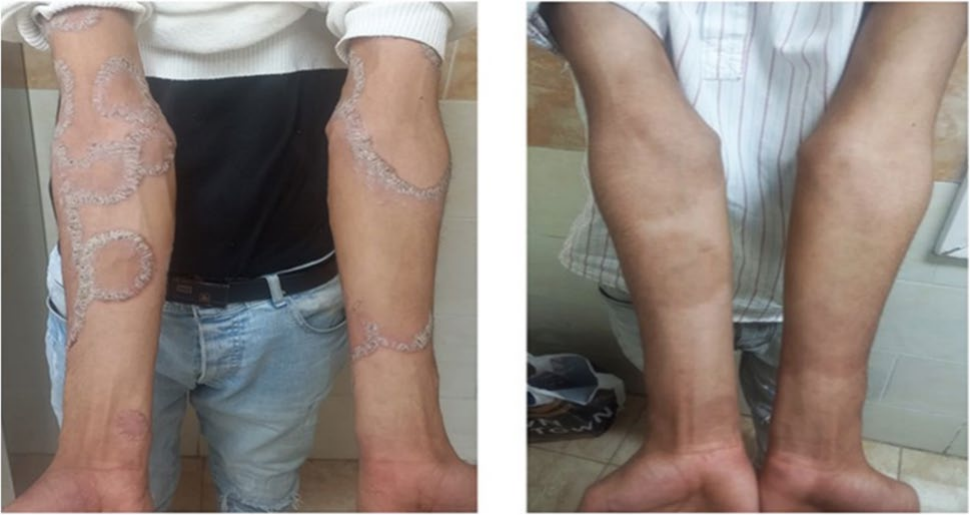
Representative case on methotrexate before and after therapy

TWEAK levels do not correlate significantly with PASI or DAPSA at baseline. After 24 weeks, TWEAK shows a moderate negative correlation with PASI (*r* =  − 0.463, *p* = 0.010) (Fig. 3), indicating an association with improvement in skin lesions. No significant correlations are observed between TWEAK and DAPSA at any time point, suggesting TWEAK reflects cutaneous rather than joint disease activity. PASI (Fig. 4) and TWEAK are both stable over time, as shown by strong baseline-to-post-treatment correlations (*r* = 0.776 and 0.812, respectively) (Table 5). Response stratification with adalimumab showed that most patients achieved good to excellent responses according to PASI response as in Fig. 5, while methotrexate has been shown to be more effective than adalimumab in treating moderate to severe plaque psoriasis, with significant improvements in skin clearance according to PASI response as in Fig. 6.

**Table 5:** Correlation between TWEAK levels and disease activity scores (PASI, DAPSA) at baseline and after treatment

Variables compared	*r*	pV
PASI baseline vs PASI after 24 weeks	0.776	< 0.001
TWEAK baseline vs TWEAK after 24 weeks	0.812	< 0.001
DAPSA baseline vs DAPSA week 24	− 0.269	0.151
TWEAK baseline vs PASI baseline	− 0.141	0.458
TWEAK after 24 weeks vs PASI after 24 weeks	− 0.463	0.010
TWEAK baseline vs DAPSA baseline	0.068	0.720
TWEAK after 24 weeks vs DAPSA after 24 weeks	0.063	0.742

*r* = Pearson correlation coefficient; *pV*, *p* value

## Discussion

The present case–control study revealed that serum tumor necrosis factor (TNF)–related weak inducer of apoptosis (TWEAK) levels were significantly elevated in psoriasis vulgaris and psoriatic arthritis (PsA) cases compared to healthy controls. Furthermore, adalimumab or methotrexate treatment for 24 weeks led to reduced TWEAK levels, together with PASI and DAPSA score improvement. The magnitudes of these reductions and their strong correlations with clinical response provide rationale for employing TWEAK as a biomarker of disease activity and drug effect in psoriatic disease [10, 12, 15, 17].

### TWEAK in psoriatic pathogenesis

TWEAK, a member of the TNF superfamily, exerts pleiotropic effects via its receptor, fibroblast growth factor–inducible 14 (Fn14), including the induction of pro-inflammatory cytokines, angiogenesis, and control of tissue remodeling [3, 4]. It is involved in autoimmune and inflammatory conditions such as rheumatoid arthritis, systemic lupus erythematosus, and inflammatory myopathies [5, 11]. Although few studies have explored its role in psoriasis and PsA, the chronic inflammatory milieu in these diseases—characterized by upregulation of IL-17, TNF-α, and IL-23—suggests the potential for TWEAK to synergize in enhancing inflammation [10, 12, 15, 17]. Our finding of elevated TWEAK in psoriasis and PsA is in agreement with previous work, where expression of TWEAK was reported to be upregulated in psoriatic skin lesions and serum and correlated with the severity of the disease [10].

### Effect of treatment on TWEAK

Both adalimumab and methotrexate significantly reduced serum TWEAK levels after 24 weeks. Patients treated with adalimumab showed a larger absolute reduction, consistent with the broad inhibitory effects of TNF-α antagonists on inflammatory mediators [6, 9]. Methotrexate also reduced TWEAK, likely through its immunosuppressive mechanisms, including inhibition of T-cell activation and cytokine release [7, 13, 14]. Together, these findings indicate that systemic therapies for psoriatic disease effectively downregulate TWEAK, suggesting it may be part of the inflammatory cascade modulated by both biologics and DMARDs. Adalimumab induced a more pronounced TWEAK reduction compared to methotrexate, likely due to direct TNF-α pathway modulation. Methotrexate’s reduction of TWEAK can be attributed to immunosuppressive and anti-cytokine effects. [9]. The observed decline in TWEAK paralleled clinical remission in skin lesions and joint activity, supporting its potential as a dynamic biomarker [6, 7, 9, 13, 14].

### Clinical response and correlation with TWEAK

Parallel to biochemical changes, psoriasis patients showed significant PASI improvement, while PsA patients demonstrated substantial DAPSA reduction [18, 19]. Our results suggest that TWEAK may play a role in psoriasis-related inflammatory pathways rather than serving as a general marker of systemic inflammation or joint disease activity. In the present study, TWEAK levels were significantly elevated in both psoriasis and PsA patients compared to controls at baseline and significantly decreased after treatment. However, no significant correlations were observed between baseline TWEAK levels and PASI or DAPSA scores, indicating that TWEAK does not directly reflect baseline disease severity. Interestingly, a significant negative correlation was detected between post-treatment TWEAK levels and PASI scores, while no such association was found with DAPSA scores either before or after treatment. This pattern suggests that TWEAK may be more indicative of treatment-related improvement in skin disease activity rather than joint involvement. Although CRP was not evaluated in this study, it is widely recognized as a marker of systemic inflammation. The lack of association between TWEAK and joint disease activity, along with its relationship to cutaneous improvement, supports the hypothesis that TWEAK may reflect local inflammatory mechanisms within the skin rather than generalized systemic inflammation. Further studies incorporating conventional inflammatory markers such as CRP are warranted to clarify the distinct inflammatory pathways represented by TWEAK in psoriasis and PsA. This aligns with evidence suggesting that circulating biomarkers can reflect therapeutic efficacy in psoriatic disease [8]. The lack of correlation between TWEAK and DAPSA underscores that this biomarker may primarily reflect epidermal rather than synovial inflammation. Our findings agree with previous literature showing elevated TWEAK expression in psoriatic skin and serum [10].

### TWEAK as a biomarker

The measurement of serum TWEAK is technically viable and reproducible in standard laboratory environments utilizing enzyme-linked immunosorbent assay (ELISA) methodologies [12]. Commercially available ELISA kits exhibit low intra- and inter-assay coefficients of variation, indicating satisfactory analytical precision [12]. TWEAK, as a circulating cytokine, demonstrates swift regulation and a comparatively brief biological half-life, enabling it to indicate dynamic alterations in inflammatory activity in response to treatment [4]. This biological behavior likely elucidates its susceptibility to treatment-induced alterations and substantiates its prospective function as a real-time biomarker of disease activity [23].

### Gender and disease response

No significant gender-related differences were detected in treatment responses for TWEAK, PASI, or DAPSA, suggesting that the biomarker behaves similarly across sexes. This observation is consistent with previous epidemiological studies that have reported comparable disease burden between males and females, despite some differences in clinical phenotype [2].

### Clinical implications

The consistent elevation and subsequent reduction of TWEAK with treatment highlight its potential as a biomarker for psoriasis monitoring. Given its correlation with PASI, serum TWEAK measurement could serve as an adjunctive tool in assessing therapeutic response, especially in cases where clinical evaluation alone may be insufficient. Furthermore, preclinical studies have shown that blocking the TWEAK/Fn14 axis reduces tissue inflammation in autoimmune models [11], suggesting a possible therapeutic avenue for psoriatic disease beyond conventional TNF-α inhibition. This study bridges a gap by concurrently assessing psoriasis and PsA within one framework, demonstrating both cutaneous and articular relevance. Despite comprehensive research endeavors, no singular validated serum biomarker has been generally implemented for routine clinical surveillance of psoriasis or psoriatic arthritis. TWEAK should not be considered the primary biomarker in psoriatic illness but rather a promising supplementary biomarker that may enhance existing clinical indices such as PASI and DAPSA. Its dynamic modulation with therapy underscores its potential utility in assessing treatment response rather than in diagnostic categorization [22].

### Strengths and limitations

Strengths of this research involve its prospective design, inclusion of both psoriasis and PsA cohorts, and standardized clinical and laboratory assessments. Limitations involve the relatively small sample size, single-center setting, and limited follow-up of 24 weeks, which limits long-term biomarker interpretation. Future studies should extend follow-up and explore mechanistic pathways linking TWEAK/Fn14 axis to psoriatic inflammation.

## Conclusion

Serum TWEAK levels are significantly elevated in psoriasis and PsA and decline after therapy with adalimumab or methotrexate. Their correlation with PASI but not DAPSA suggests that TWEAK better mirrors cutaneous disease activity. TWEAK may represent a useful adjunct biomarker for monitoring treatment response in psoriatic disease and for disease monitoring and a potential future therapeutic target in psoriatic disease.

## Data Availability

Available from the corresponding author upon reasonable request.

## Abbreviations

*DAPSA*: Disease Activity Index for Psoriatic Arthritis
*ELISA*: Enzyme‑linked immunosorbent assay
*Fn14*: Fibroblast growth factor–inducible 14
*PASI*: Psoriasis Area and Severity Index
*PsA*: Psoriatic arthritis
*TWEAK*: TNF‑related weak inducer of apoptosis
*TNF‑α*: Tumor necrosis factor‑alpha
